# Automated identification of incidental hepatic steatosis on Emergency Department imaging using large language models

**DOI:** 10.1097/HC9.0000000000000638

**Published:** 2025-02-19

**Authors:** Tyrus Vong, Nicholas Rizer, Vedant Jain, Valerie L. Thompson, Mark Dredze, Eili Y. Klein, Jeremiah S. Hinson, Tanjala Purnell, Stephen Kwak, Tinsay Woreta, Alexandra T. Strauss

**Affiliations:** 1 Division of Gastroenterology & Hepatology, Johns Hopkins University School of Medicine, Baltimore, Maryland, USA; 2 Department of Emergency Medicine, Johns Hopkins University School of Medicine, Baltimore, Maryland, USA; 3 Department of Gastroenterology & Hepatology Carle Illinois College of Medicine, University of Illinois at Urbana-Champaign, Urbana, Illinois, USA; 4 Department of Computer Science, Johns Hopkins University, Baltimore, Maryland, USA; 5 Department of Epidemiology, Johns Hopkins University Bloomberg School of Public Health, Baltimore, Maryland, USA; 6 Department of Radiology, Johns Hopkins University School of Medicine, Baltimore, Maryland, USA

**Keywords:** fatty liver, OpenAI, radiology

## Abstract

**Background:**

Hepatic steatosis is a precursor to more severe liver disease, increasing morbidity and mortality risks. In the Emergency Department, routine abdominal imaging often reveals incidental hepatic steatosis that goes undiagnosed due to the acute nature of encounters. Imaging reports in the electronic health record contain valuable information not easily accessible as discrete data elements. We hypothesized that large language models could reliably detect hepatic steatosis from reports without extensive natural language processing training.

**Methods:**

We identified 200 adults who had CT abdominal imaging in the Emergency Department between August 1, 2016, and December 31, 2023. Using text from imaging reports and structured prompts, 3 Azure OpenAI models (ChatGPT 3.5, 4, 4o) identified patients with hepatic steatosis. We evaluated model performance regarding accuracy, inter-rater reliability, sensitivity, and specificity compared to physician reviews.

**Results:**

The accuracy for the models was 96.2% for v3.5, 98.3% for v4, and 98.8% for v4o. Inter-rater reliability ranged from 0.99 to 1.00 across 10 iterations. Mean model confidence scores were 2.9 (SD 0.8) for v3.5, 3.9 (SD 0.3) for v4, and 4.0 (SD 0.07) for v4o. Incorrect evaluations were 76 (3.8%) for v3.5, 34 (1.7%) for v4, and 25 (1.3%) for v4o. All models showed sensitivity and specificity above 0.9.

**Conclusions:**

Large language models can assist in identifying incidental conditions from imaging reports that otherwise may be missed opportunities for early disease intervention. Large language models are a democratization of natural language processing by allowing for a user-friendly, expansive analyses of electronic medical records without requiring the development of complex natural language processing models.

## INTRODUCTION

Hepatic steatosis is a common disease and known precursor to severe liver disease with high morbidity and mortality.^1^ The prevalence of steatotic liver disease between 2017 and 2020 was estimated to be 37.87%.^2^ Hepatic steatosis is typically seen on imaging such as CT, MRI, or ultrasound.^3^ In the Emergency Department (ED), abdominal imaging frequently occurs as part of routine patient care for work-up of other acute issues.^4^ However, incidental findings of hepatic steatosis in imaging reports are not translated to further testing, diagnoses, and treatment due to the appropriate focus at the time on the acute concern of that ED encounter.^2^ For example, an ED physician concerned about appendicitis is appropriately focused on the part of the report about the appendix and not the incidental fatty liver.

Radiology reports contain a wealth of information that is not readily available as discrete data. Extraction of useful information from these fields requires natural language processing (NLP). Researchers have created NLP algorithms to identify hepatic steatosis and other liver diseases, but had limitations of needing to define key terms and negation cases and is resource intensive, such as needing computer scientists and expertise with coding for development and validation.^5^,^6^ These traditional NLP systems require custom code development and labeling for each specific task and make actual implementation and general application difficult. An alternative approach relies on large language models (LLMs) that are designed as general-purpose tools for language tasks.^7^ LLMs like OpenAI’s Generative Pretrained Transformer (GPT) models were trained on extensive text samples from the internet and other sources enabling them to understand and generate human language.^8^,^9^ LLMs had already demonstrated promise in other medical applications,^10^,^11^ suggesting their basic ability to process clinical text. ChatGPT serves as the conversational interface for OpenAI’s GPT models, which, when provided a detailed set of instructions that describe the task (a prompt) and a clinical text record, could automatically process entire imaging reports quickly and without clinical situational bias.

Using abdominal imaging reports for ED patients, we evaluated the performance of 3 LLM Azure OpenAI models: 3.5, 4, and 4o. to determine the presence of hepatic steatosis. We also assessed the LLM’s ability to be reliable and to describe confidence with responses. We hypothesized that new LLMs could perform tasks, such as early detection of hepatic steatosis, without intensive NLP expertise, with high reliability, and with high interpretability. This study is important for understanding: (1) how LLMs can assist in identifying incidental medical conditions, such as hepatic steatosis, for improved early disease detection and intervention, and (2) the more user-friendly potential of LLMs as an expansive analysis of electronic medical records without requiring the development of complex NLP models from scratch.

## METHODS

### Study population

We included adults who had undergone CT abdominal imaging performed in the ED between August 1, 2016, and December 31, 2023, at 1 of 5 hospitals within our health system (IRB00404419). These hospitals serve a diverse patient population and encompass both academic and community sites in urban and nonurban settings. Physicians from hepatology (Alexandra T. Strauss) and emergency medicine (Nicholas Rizer) labeled CT reports for evidence of hepatic steatosis (100) or not (100) for a total data set of 200. They also indicated their level of confidence for each label on a scale of “not confident at all” to “very confident.” The physician's answers were converted to a scale of 1 to 4, where 1 was “not confident at all,” 2 was “somewhat not confident,” 3 was “somewhat confident,” and 4 was “very confident.”^12^,^13^ They reconciled discrepancies through consensus and a radiologist (Stephen Kwak) was available for adjudication as needed. We selected CTs because they were the most common abdominal imaging procedures performed in the ED and have higher odds of incidental liver findings compared to ultrasound.^14^ We chose the sample size of 200 based on an electronic health record phenotype validation method from Liu et al.^15^ To ensure a fair and equitable data set, we used quota sampling and selected an equal distribution of patients based on race, ethnicity, and sex. We randomly sampled these groups until we had a data set of 100 positive reports and 100 negative reports. We collected additional patient data commonly observed in the ED setting such as sex, age, ethnicity, race, body mass index, laboratory results, and comorbidities, such as ICD-10 codes for hypertension, type 2 diabetes, hyperlipidemia, obesity, chronic kidney disease, and metabolic dysfunction–associated steatotic liver disease. We calculated Fibrosis-4 (FIB-4), an index to predict fibrosis, using AST, AST, and platelet.^16^ The lab results used to calculate the FIB-4 were the lab results drawn closest to the time of arrival in the ED within 6 months.

### Data processing

Notes from imaging reports contain non-clinically relevant text and some partially structured text. For preprocessing, we identified structured sections of the note using known phrase patterns (eg, “indication:, findings:”) to mark the location of the relevant narrative or impression section. For relevant sections, we extracted the text identified for that section until the next section, treating each phrase pattern as a section. The phrase pattern for “liver:” did not always appear as a section within reports, even if the narrative/impression mentioned liver findings. To address this, we identified the phrases “liver,” “hepatic,” or “hepatitis” within the text and returned the text from 1 section before and 1 section after. If we did not identify any text specifically mentioning the liver, we returned the entire narrative and impression.

### Prompt engineering and model settings

**FIGURE 1:**
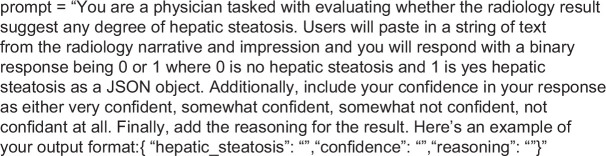
Prompt ChatGPT to evaluate whether a radiology report was suggestive of hepatic steatosis.

The prompt asked the LLM to identify whether the imaging report suggested that the patient had hepatic steatosis. It also asked the LLM to return its answer as a binary 1 or 0, provide confidence in its answer, and explain its reasoning (Figure 1). We asked the LLM to provide its answers in the same manner as the physicians as previous literature demonstrated the ability to provide its uncertainty in words.^13^ We tested the temperature parameter, which controls the model’s randomness (a lower temperature indicates less random text generation), at both 0 and 1.^17^ For the final prompt, we set the temperature to 0 and disabled the violence filter to allow for the processing of medically oriented reports. For example, we found some typical ED encounters related to traumas would fail to run through the GPT model due to censoring from words like “bullets,” “gunshot,” and “knife.” We also tested a few-shot prompt in which 2 positive examples and 2 negative examples were provided (Supplemental Figure S1, http://links.lww.com/HC9/B903).

### Analysis

We inputted the notes into model versions 3.5 (version 0613), 4 (version turbo-2024-04-09), and 4o (version 2024-05-13) for 10 iterations. We used a HIPAA-compliant version of the models available through Azure. Compared to our physician-determined gold standard, we calculated accuracy, Cohen’s kappa, sensitivity, specificity, positive predictive value (PPV), and negative predictive value (NPV) for each model. We assessed the confidence for each model. Due to a certain degree of randomness to GPT model outputs, we aimed to assess trustworthiness by performing multiple iterations per model with the same data. We calculated inter-rater reliability between 10 iterations of each model by calculating Cohen’s kappa (ie, treating each iteration on a model as a different “rater” and comparing 10 raters on that model). We calculated the cost per note using the pricing of OpenAI models as of November 5, 2024.^18^ We performed all analyses using Python 3 version 3.10.11.

## RESULTS

### Study population

The study included 101 (50.5%) males and 99 (49.5%) females with a median age of 42 (Table 1). The patients’ race and ethnicity were 50 (25.0%) Non-Hispanic White, 50 (25.0%) Non-Hispanic Black, 51 (25.5%) Hispanic or Latino, and 49 (49.5%) Non-Hispanic Other. There were 52 (26%) patients with hypertension, 28 (14.0%) with type 2 diabetes, 23 (11.5%) with hyperlipidemia, 19 (9.5%) with obesity, and 7 (3.5%) with chronic kidney disease. Patients had a median body mass index of 28.08 (24.24, 32.78). Patients had a median ALT of 28, a median AST of 25, and a median platelet of 262. There were 121 (60.5%) patients with low-risk (FIB-4 <1.3) FIB-4, 49 (24.5%) patients with high-risk FIB-4 (FIB-4 ≥1.3), and 30 (15%) patients with unknown FIB-4. There were 2 (1.0%) patients with a prior diagnosis of metabolic dysfunction–associated steatotic liver disease and 4 (2.0%) patients with a follow-up diagnosis of metabolic dysfunction–associated steatotic liver disease. There were 13 (6.5%) patients who had a follow-up visit with a gastroenterology or hepatology clinic.

**TABLE 1:** Characteristics of patients evaluated in the Emergency Department with CT

Characteristics	Overall (N=200)
Age, median (IQR)	42 (32, 59.5)
Sex, female, n (%)	
Male	101 (50.5)
Race and ethnicity, n (%)	
Non-Hispanic White	50 (25.0)
Non-Hispanic Black	50 (25.0)
Hispanic or Latino	51 (25.5)
Non-Hispanic Other	49 (24.5)
Comorbidities, n (%)	
Hypertension	52 (26.0)
Type 2 diabetes	28 (14.0)
Hyperlipidemia	23 (11.5)
Obesity	19 (9.5)
Chronic kidney disease	7 (3.5)
BMI, median (IQR)	28.08 (24.24,32.78)
Laboratory values	
ALT, median (IQR)	28 (17.25,54)
AST, median (IQR)	25 (19,37)
Platelets, median (IQR)	262 (210,307)
FIB-4 score, n (%)^a^	
Low risk (<1.3)	121 (60.5)
High risk (>1.3)	49 (24.5)
MASLD, n (%)	
Prior diagnosis	2 (1.0)
Follow-up diagnosis	4 (2.0)
Follow-up gastroenterology or hepatology visit, n (%)	13 (6.5)

^a^ FIB-4: 30 (15%) missing lab(s) needed to calculate FIB-4.

Abbreviations: BMI, body mass index; FIB-4, fibrosis-4; MASLD, metabolic dysfunction–associated steatotic liver disease.

### Classification performance

**FIGURE 2:**
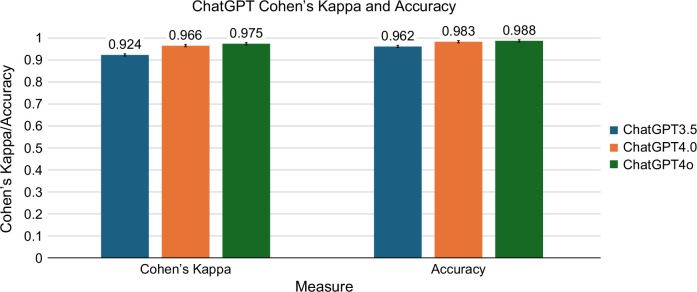
Evaluation of the accuracy of GPT models.

**FIGURE 3:**
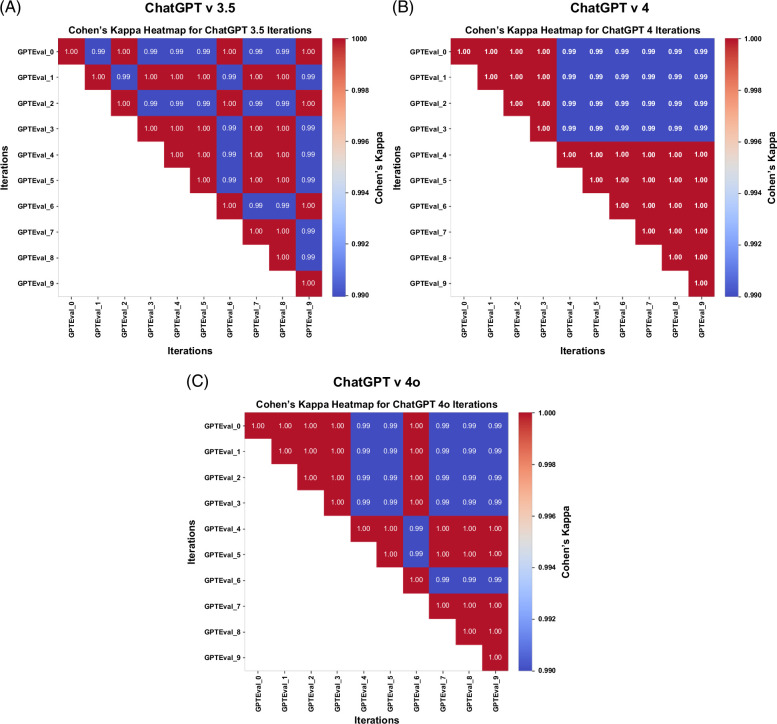
Inter-rater reliability per ChatGPT version. The inter-rater reliability between ten different iterations was accessed for each ChatGPT version. (A) Heatmap of the Cohen’s Kappa correlations for interrater reliability between each ChatGPT v 3.5 iteration. (B) Heatmap of the Cohen’s Kappa correlations for interrater reliability between each ChatGPT v 4 iteration. (C) Heatmap of the Cohen’s Kappa correlations for interrater reliability between each ChatGPT v 4o iteration. The color scale ranges from blue to red, where blue indicates lower agreement and red indicates higher agreement. A Cohen’s Kappa value closer to 1 indicates high agreement, while a value close to 0 reflects weak agreement.

Compared to the physician assessment gold standard, the average accuracy for model v3.5 was 96.2% and a Cohen’s kappa of 0.924. The average accuracy for v4 was 98.3% and a Cohen’s kappa of 0.966. The average accuracy for v4o was 98.8% with a Cohen’s kappa of 0.975. (Figure 2) For v3.5, the sensitivity was 0.994, the specificity was 0.929, the PPV was 0.935, and the NPV was 0.994 (Table 2). For v4, the sensitivity was 0.966, the specificity was 1, the PPV was 1, and the NPV was 0.967. For v4o, the sensitivity was 0.975, the specificity was 1, the PPV was 1, and the NPV was 0.975. When we tested each model’s consistency with 10 iterations, all 3 models showed high (0.99–1.00) inter-rater reliability between iterations (Figure 2) (Figure 3). The estimated cost per 100 reports for v3.5 was $0.02 (IQR: 0.02, 0.03) (Supplemental Table S1, http://links.lww.com/HC9/B905). The estimated cost per 100 reports for v4 was $1.35 (IQR: 1.09, 1.67). The estimated cost per 100 reports for v4o was $0.24 (IQR: 0.19, 0.30). In the few-shot prompt, the average accuracy for model v3.5 was 98.7% and a Cohen’s kappa of 0.973 (Supplemental Figure S2, http://links.lww.com/HC9/B904). The average accuracy for v4 was 98.6% and a Cohen’s kappa of 0.971. The average accuracy for v4o was 99.8% with a Cohen’s kappa of 0.996.

**TABLE 2:** Performance characteristics of ChatGPT in identifying hepatic steatosis

Model	Sensitivity	Specificity	Positive predictive value	Negative predictive value
ChatGPT v 3.5	0.99	0.93	0.94	0.99
ChatGPT v 4	0.97	1	1	0.97
ChatGPT v 4o	0.98	1	1	0.98

### Confidence

**FIGURE 4:**
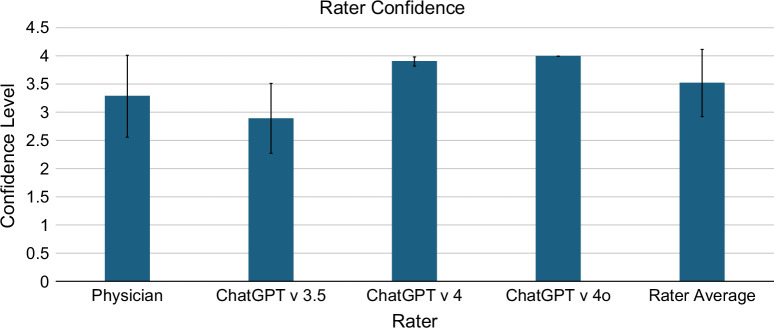
Overall confidence of the rater in its evaluation of reports for hepatic steatosis.

We evaluated the rater’s overall confidence on a scale of 1–4, where 1 was “not confident at all,” 2 was “somewhat not confident,” 3 was “somewhat confident,” and 4 was “very confident.” The mean physician confidence was 3.3 (SD: 0.9) (Figure 4). The mean confidence of Azure OpenAI v3.5 was 2.9 (SD: 0.8). The mean confidence of Azure OpenAI v4 was 3.9 (SD: 0.3). The mean confidence of Azure OpenAI v4o was 4.0 (SD: 0.07). The mean rater confidence across the physicians and GPT models was 3.5 (SD: 0.6).

### Incorrect evaluations

We evaluated the models on the imaging reports they got incorrect. Of the 76 (3.8%) incorrect labels of model v3.5, the range count of incorrect across iterations per imaging report was 6–10, the mean count of incorrect across iterations per imaging report was 9.5 (SD: 1.4), and the mean model confidence was 3 (SD: 0) (Supplemental Table S1, http://links.lww.com/HC9/B905). Of the 34 (1.7%) incorrect labels of model v4, the range count of incorrect across iterations per imaging report was 4–10, the mean count of incorrect across iterations per imaging report was 8.5 (SD: 3.0), and the mean model confidence was 3 (SD: 0). Of the 25 (1.3%) incorrect labels of model v4o, the range count of incorrect across iterations per imaging report was 5–10, the mean count of incorrect across iterations per imaging reports was 8.3 (SD: 2.9), and the mean model confidence was 4 (SD: 0).

We then evaluated the rationale of the models on the imaging reports they got incorrect. For incorrect answers of model v3.5, 0 (0%) was attributed to focal fat, 5 (62.5%) was attributed to mild steatosis, 2 (25.0%) was attributed to attenuation or enhancement, 1 (12.5%) was attributed to grammatical errors (Supplemental Table S2, http://links.lww.com/HC9/B906). For incorrect answers of model v4, 4 (100%) was attributed to fat in the local or adjacent area, 0 (0%) was attributed to mild steatosis, 0 (0%) was attributed to attenuation or enhancement, and 0 (0%) was attributed to grammatical errors. For incorrect answers of model v4o, 3 (100%) was attributed to fat in the local or adjacent area, 0 (0%) was attributed to mild steatosis, 0 (0%) was attributed to attenuation or enhancement, and 0 (0%) was attributed to grammatical errors. For the few-shot approach, incorrect answers were due to attenuation (v3.5: 1 [33.3%]), focal fat (v3.5: 1 [33.3%]; v4: 3 [100%]; v4o: 1 [100%]), and mild steatosis (v3.5: 1 [33.3%]).

## DISCUSSION

In this study, we demonstrated that LLMs can classify free-text radiology reports of ED patients as suggestive of hepatic steatosis with a high degree of accuracy (>95%). Each model (OpenAI GPT v3.5, 4, 4o) demonstrated high inter-rater reliability, which showed consistency across iterations on the same imaging reports. The models exhibited high agreement with physician review and increased agreement with each model upgrade (3.5>4>4o).

The method we defined in this study demonstrated the relative ease of implementing an LLM to identify hepatic steatosis in imaging, showing that an LLM could categorize complex conditions from imaging reports without the need for NLP experts and extensive coding. Prior studies have demonstrated that custom NLP models have the ability to identify hepatic steatosis and other liver diseases from imaging reports.^5^,^6^ Validation and training of these models required multiple rounds of queries to identify key terms and negation cases, text processing, manual chart review, and algorithm assessment, which is time-consuming and requires time from both NLP experts and physicians. However, our study utilized off-the-shelf LLMs, which are pretrained to understand human language and can be done by non-NLP experts using the conversational ChatGPT interface to the OpenAI LLM models. Another advantage of using off-the-shelf LLMs was that they were readily available, requiring only time for prompt engineering rather than data annotation and model training, which are time-intensive.

In our analysis of the imaging reports that were incorrect in identifying hepatic steatosis, we found that the expressed model confidence is not correlated with the accuracy of the model. Even when incorrect, the model confidence was between “somewhat confident” and “very confident” in its answers across all models. Prior studies have shown that model accuracy and confidence are not always well calibrated, but there are methods to improve the correlation between model accuracy and confidence.^12^,^19^ In the cases that were identified incorrectly by the model, we were able to identify the reasoning for its answer. For model v3.5, there were a variety of reasons for incorrect answers including misclassification of mild or moderate steatosis, attenuation or enhancement, or report punctuation. There was a single report where punctuation was missing between describing a hepatic cyst/adenoma and hepatic steatosis, and the model only reasoned about the first finding. Conversely, the only theme in the incorrect cases for model v4 and model v4o was determining focal fat (eg, falciform ligament) was hepatic steatosis. The differences in themes for incorrect answers between models may be due to improvements in language comprehension in subsequent models allowing for improved classification of less explicit reports. Using a few-shot prompt, there was a slight improvement in accuracy and similar reasons for incorrect responses. Utilizing a general-purpose LLM for chart review improved research transparency, as the input and output of the model are humanly interpretable, allowing us to understand the rationale for its answers and providing insight on how we can further improve our prompt.

Our study demonstrated that multiple GPT models had the ability to identify incidental findings with high accuracy. Our finding supports previous literature that highlighted the ability of GPT v4 to identify actionable incidental findings in the ED setting.^20^ Our study focuses on identifying incidental hepatic steatosis in the ED setting and adds to the growing potential use cases of LLMs in liver disease, as seen in prior studies using LLMs to extract information from imaging reports of patients with HCC.^21^ A potential use case is early detection where the imaging reports are reviewed automatically by a model which could help identify patients with hepatic steatosis who might otherwise be missed. There are prior studies that highlight the increased need for identifying patients who have potentially life changing conditions earlier. According to a meta-analysis study in 2022, 31% of CTs had incidental findings on CT imaging, which demonstrated the need for identifying patients with underlying conditions earlier.^22^ Unlike our study, these patients had to be identified by manual chart review and was not performed in an automated fashion as in our study. The findings of our study could be utilized to help identify patients who need follow-up care without increasing physician burden through manual chart review.

A limitation of our study was being a single institution study. However, we included data from 5 diverse EDs in urban/rural and academic/community settings. In addition, we used publicly available models, which support the validation of our findings at other institutions. While one could develop a custom NLP model for a single setting, the general-purpose LLM we used could facilitate easier adoption at multiple institutions. One limitation of our study was that we did not have tissue confirmation of hepatic steatosis as this was outside the scope of this study. In this study, we were not able to assess whether patients had a linkage to follow-up care, but it could be an avenue for future studies. For future studies, we will consider the use of LLMs for improving connecting ED patients with potential liver disease to outpatient care.

In conclusion, our findings demonstrated that LLMs can label imaging reports suggestive of hepatic steatosis with high accuracy compared to physician review. This methodology demonstrates the potential to screen and identify patients in the ED who may be at risk of hepatic steatosis from incidental liver findings. These findings demonstrate that LLMs can assist in screening for incidental medical conditions from imaging reports that otherwise may be missed opportunities for early disease detection and intervention. Overall, LLMs offer a readily available solution to identifying patients at risk of underdiagnosed medical conditions, such as hepatic steatosis, without the development and training time needed for traditional NLP.

## Supplementary Material
