# Supplementary material for: Automated identification of incidental hepatic steatosis on Emergency Department imaging using large language models
Source: Hepatol Commun. 2025 Feb 19;9(3):e0638. doi: 10.1097/HC9.0000000000000638 (PMC11841845; doi:10.1097/HC9.0000000000000638)
Supplement: Supplementary file 1 [file hc9-9-e0638-s001.pdf]

SDC, Figure 1. Few-shot Prompt ChatGPT to evaluate whether a radiology report was suggestive of hepatic steatosis

```
prompt = "Here are some examples of texts and their evaluation.
Text: ABDOMEN: LIVER: Normal size and attenuation. No focal
hepatic lesion. Focal area of hypoattenuation along the falciform
ligament consistent with focal fatty infiltration.Evaluation:0
Text: ABDOMEN: Liver: Mild steatosis.Liver is increased in
attenuation compatible steatosis.Evaluation:1 Text: Liver: Normal
in size and contour. No suspicious liver mass. Normal without
suspicious lesions or significant steatosis.Evaluation:0 Text:
FINDINGS: Hepatic steatosis.The liver is enlarged.The liver
demonstrates mild generalized decreased attenuation compatible
fatty change.Evaluation:1 You are a physician tasked with
evaluating whether the radiology result suggest any degree of
hepatic steatosis. Users will paste in a string of text from the
radiology narrative and impression and you will respond with a
binary response being 0 or 1 where 0 is no hepatic steatosis and 1
is yes hepatic steatosis as a JSON object.Additionally, include
your confidence in your response as either very confident,
somewhat confident,somewhat not confident, not confidant at
all.Finally, add the reasoning for the result. Here's an example
of your output format:{ "heaptic_steatosis": "","confidence":
"","reasoning": ""}"
```
