# Supplementary figures and images for: Automated identification of incidental hepatic steatosis on Emergency Department imaging using large language models
Source: Hepatol Commun. 2025 Feb 19;9(3):e0638. doi: 10.1097/HC9.0000000000000638 (PMC11841845; doi:10.1097/HC9.0000000000000638)

**SDC, Figure 2. Cohen's Kappa and Accuracy of Few-shot Prompt**

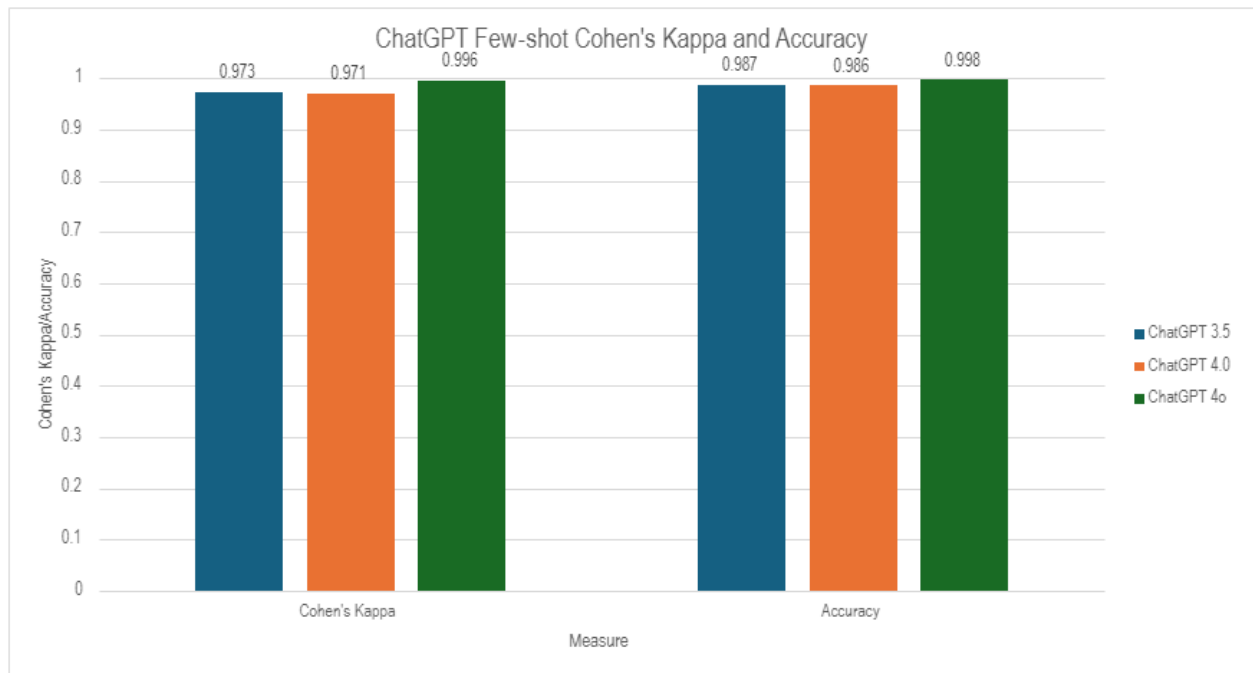

Supplement: Supplementary file 2 [file hc9-9-e0638-s002.pdf]
