# Supplementary material for: Automated identification of incidental hepatic steatosis on Emergency Department imaging using large language models
Source: Hepatol Commun. 2025 Feb 19;9(3):e0638. doi: 10.1097/HC9.0000000000000638 (PMC11841845; doi:10.1097/HC9.0000000000000638)
Supplement: Supplementary file 3 [file hc9-9-e0638-s003.docx]

**SDC, Table 1. Estimated cost for each ChatGPT Model**

|  |  | **Model** |  |
| --- | --- | --- | --- |
| **Item** | **ChatGPT v3.5** | **ChatGPT v4** | **ChatGPT v4o** |
| Input token number | 363 (298,446) | 363 (298,446) | 355 (291,435) |
| Output token number | 43 (32,55) | 43 (32,55) | 41 (30,53) |
| Cost per input token* | $0.50/1M tokens | $30/1M tokens | $5/1M tokens |
| Cost per output token* | $1.50/1M tokens | $60/1M tokens | $15/1M tokens |
| Estimated cost per 100 reports | $0.02 (0.02,0.03) | $1.35 (1.09,1.67) | $0.24 (0.19,0.30) |

Results reported as median (IQR) except as otherwise noted

*Based on ChatGPT OpenAI Pricing

Abbreviations: IQR, Interquartile Range
