# Supplementary material for: Automated identification of incidental hepatic steatosis on Emergency Department imaging using large language models
Source: Hepatol Commun. 2025 Feb 19;9(3):e0638. doi: 10.1097/HC9.0000000000000638 (PMC11841845; doi:10.1097/HC9.0000000000000638)
Supplement: Supplementary file 4 [file hc9-9-e0638-s004.docx]

**SDC, Table 2. Characteristics of incorrect ChatGPT evaluations.**

| **Model** | **ChatGPT v3.5** | **ChatGPT v 4** | **ChatGPT v 4o** |
| --- | --- | --- | --- |
| Incorrect of 2000 iterations | 76 (3.8%) | 34 (1.7%) | 25 (1.3%) |
| Confidence of incorrect, mean (SD)* | 3 (0) | 3 (0) | 4 (0) |
| CTs with at least 1 incorrect iteration (n=200) | 8 | 4 | 3 |
| Of incorrect CTs, range of incorrect iterations | 6-10 | 4-10 | 5-10 |
| **Rationale for Incorrectness**** |  |  |  |
| Focal fat | 0 (0%) | 4 (100%) | 3 (100%) |
| Mild steatosis | 5 (62.5%) | 0 (0%) | 0 (0%) |
| Attenuation or enhancement | 2 (25.0%) | 0 (0%) | 0 (0%) |
| Report punctuation | 1 (12.5%) | 0 (0%) | 0 (0%) |

Data reported above as N (%) unless otherwise stated. The 200 computed tomography reports were run through 10 iterations on each ChatGPT model.

*Confidence scale: 1 was “not confident at all”, 2 was “somewhat not confident”, 3 was “somewhat confident”, and 4 was “very confident”

**Report characteristics among incorrect CTs. Total number for ChatGPT v3.5 is 8, v4 is 4, v4o is 3.

Abbreviations: CT, computed tomography; SD, Standard Deviation
